# Supplementary material for: Metagenome-Based Exploration of Bacterial Communities Associated with Cyanobacteria Strains Isolated from Thermal Muds
Source: Microorganisms. 2022 Nov 25;10(12):2337. doi: 10.3390/microorganisms10122337 (PMC9785279; doi:10.3390/microorganisms10122337)

# Supplementary figure S1

**PMC 877.14**
***Planktothricoides raciborskii***

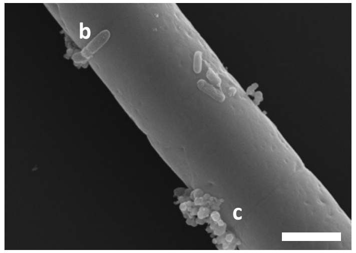


**PMC 878.14**
***Laspinema* sp.**

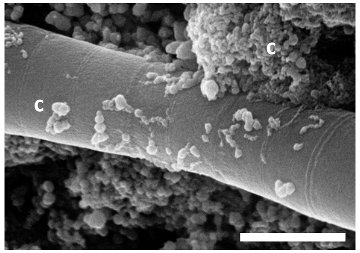


**PMC 879.14**
***Microcoleus vaginatus***

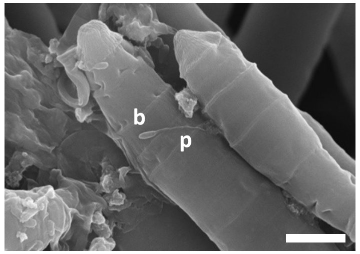


**PMC 880.14**
***Lyngbya martensiana***

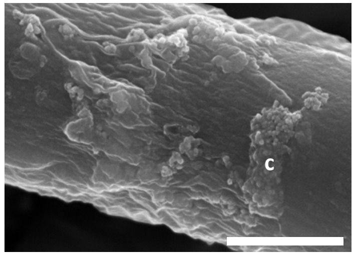


**PMC 881.14**
***Nostoc* sp.**

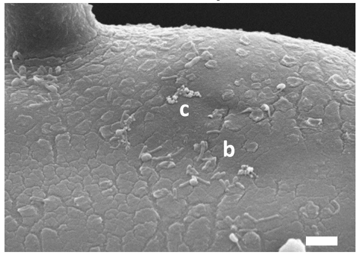


**PMC 882.14**
***Aliinostoc* sp.**

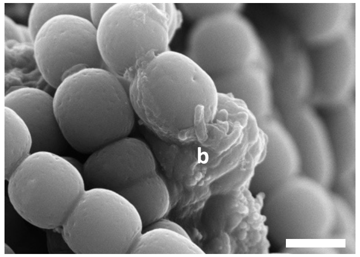


**PMC 883.14**
***Leptolyngbya boryana***

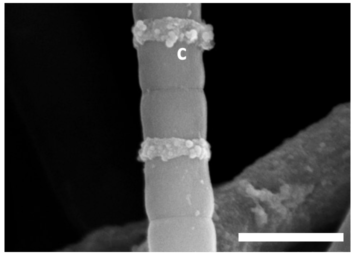


**PMC 884.14**
***Dulcicalothrix* sp.**

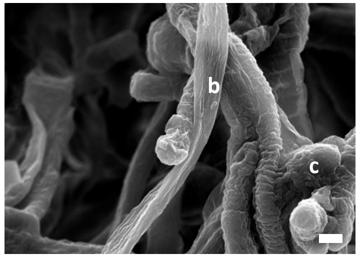


**PMC 885.14**
***Pseudochroococcus coutei***

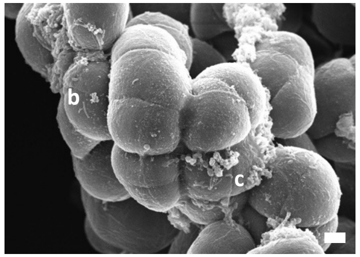

# Supplementary figure S2

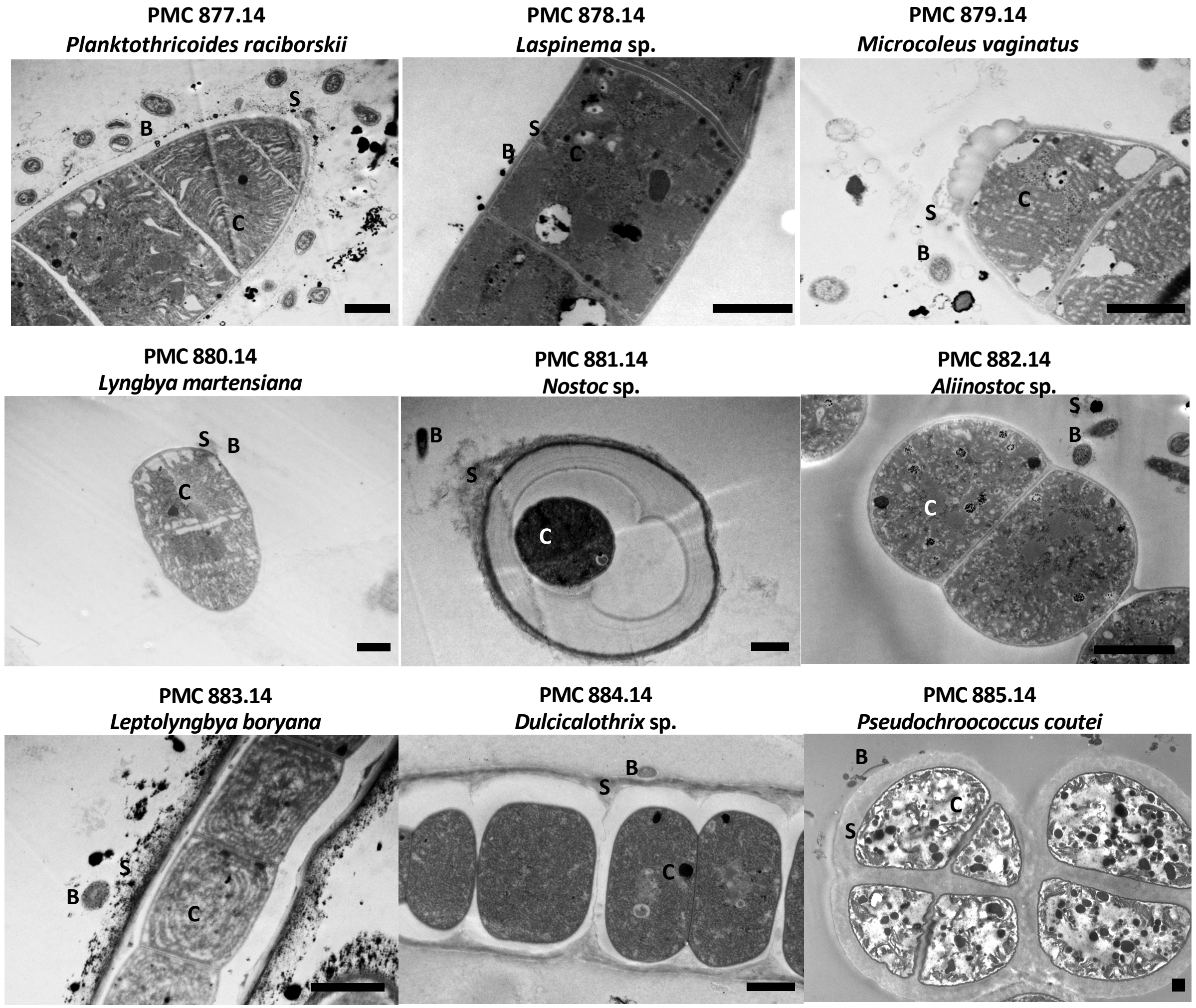

Supplement: Supplementary file 1 [file microorganisms-10-02337-s001.zip › Supp mat/Supp figS1-2.pdf]
